# Clinical observation of a modified technique for intrascleral fixation of flanged three-piece foldable intraocular lenses through a Hoffman pocket

**DOI:** 10.3389/fmed.2024.1382100

**Published:** 2024-03-13

**Authors:** Hongfei Ye, Mengxiao Wu, Wan Sun, Jiao Lyu, Yu Xu, Ping Fei, Jie Peng, Haiying Jin, Peiquan Zhao

**Affiliations:** ^1^Department of Ophthalmology, Xinhua Hospital Affiliated to Shanghai Jiaotong University School of Medicine, Shanghai, China; ^2^Department of Ophthalmology, Shanghai East Hospital, Tongji University School of Medicine, Shanghai, China

**Keywords:** three-piece intraocular lens, scleral fixation, Hoffman pockets, flange, knotting

## Abstract

**Purpose:**

To present the outcomes of a new technique for intrascleral fixation of a flanged three-piece foldable intraocular lens (IOL) without a conjunctival incision.

**Materials and methods:**

We retrospectively reviewed a consecutive series of 12 eyes of 12 patients who underwent scleral IOL fixation using this technique.

**Results:**

The follow-up period ranged 3–12 months. There was a significant improvement in best-corrected visual acuity, from 0.8 (1.6) logarithm of the minimum angle of resolution (logMAR) preoperatively to 0.45 (0.8) logMAR at the final postoperative follow-up (*p* = 0.012). Notable complications included one case of pupillary IOL capture and increased intraocular pressure.

**Conclusion:**

Our novel technique is a viable solution for managing secondary IOL fixation, enabling the use of a wider variety of IOLs and simplifying the reposition process for dislocated three-piece IOLs. This approach has the potential to lower complication rates and enhance patients’ recovery.

## Introduction

Scleral fixation of posterior chamber intraocular lens (PCIOLs) is a popular technique for secondary IOL implantation in the absence of capsular support, which allows for IOL placement in the physiologic anatomic position and theoretically mitigates complications (1). Although sutureless scleral fixation techniques can mitigate the risk of complications associated with sutures, there remains a significant concern regarding the stability of IOLs, with generally unsatisfactory long-term outcomes (2). Thus far, scleral suture fixation of PCIOLs has demonstrated superior long-term durability and offers a comparatively simpler approach to manage potential surgical complications (1, 3).

Traditional sclerally-sutured IOLs, such as CZ70BD (Alcon Laboratories, Fort Worth, Texas, USA), are composed of polymethyl methacrylate (PMMA) and require a larger incision. This variety of IOLs have become less popular over recent years given the associated risks of intraoperative damages and postoperative complications (4). Though the literatures have reported on the use of foldable IOLs for suture fixation, these studies were mostly designed with closed-loop haptics (5, 6). The option of using foldable IOLs remains limited for scleral fixation. In our previous study, a modified technique for scleral suture fixation of a three-piece PCIOL was proposed (7). While it is a general trend to develop novel fixation techniques for more flexible usage of various designs of IOLs.

In this study, we present observation outcomes of a modified technique, combining the Hoffman pocket, flanged IOL fixation technique, and a special knotting method for firm suturing and secure IOL fixation. This technique allows for flexible usage of three-piece PCIOLs under various conditions. For IOL dislocation cases, this method allows IOL reposition without the procedures of IOL explantation and reimplantation, which is highly effective and leads to a significant reduction in both intraoperative and postoperative complications. Below, we describe the utilization of this technique, its applications, and corresponding clinical results.

## Materials and methods

This study was approved by the Ethical Review Board of Xinhua Hospital Affiliated to Shanghai Jiao Tong University School of Medicine and adhered to the principles of the Declaration of Helsinki. A retrospective chart review was performed consisting of 12 eyes of 12 patients who underwent this modified scleral suture fixation of a three-piece PCIOL [Tecnis ZA9003 (Johnson and Johnson, Santa Ana, California, USA) or MA60AC (Alcon Laboratories, Fort Worth, Texas, USA)] between January 2022 and December 2022. Patients were followed up for at least 3 months after surgery. Medical records included surgical indications, relevant ocular and systemic histories, and postoperative complications were collected. All patients had undergone ophthalmic evaluations including best-corrected visual acuity (BCVA), intraocular pressure (IOP), refraction, slit-lamp biomicroscopy, dilated fundus examination, optical coherence tomography measurement, axial length, and corneal endothelial density (ECD) preoperatively and at the last visit postoperatively.

### Surgical technique

**Figure 1:**
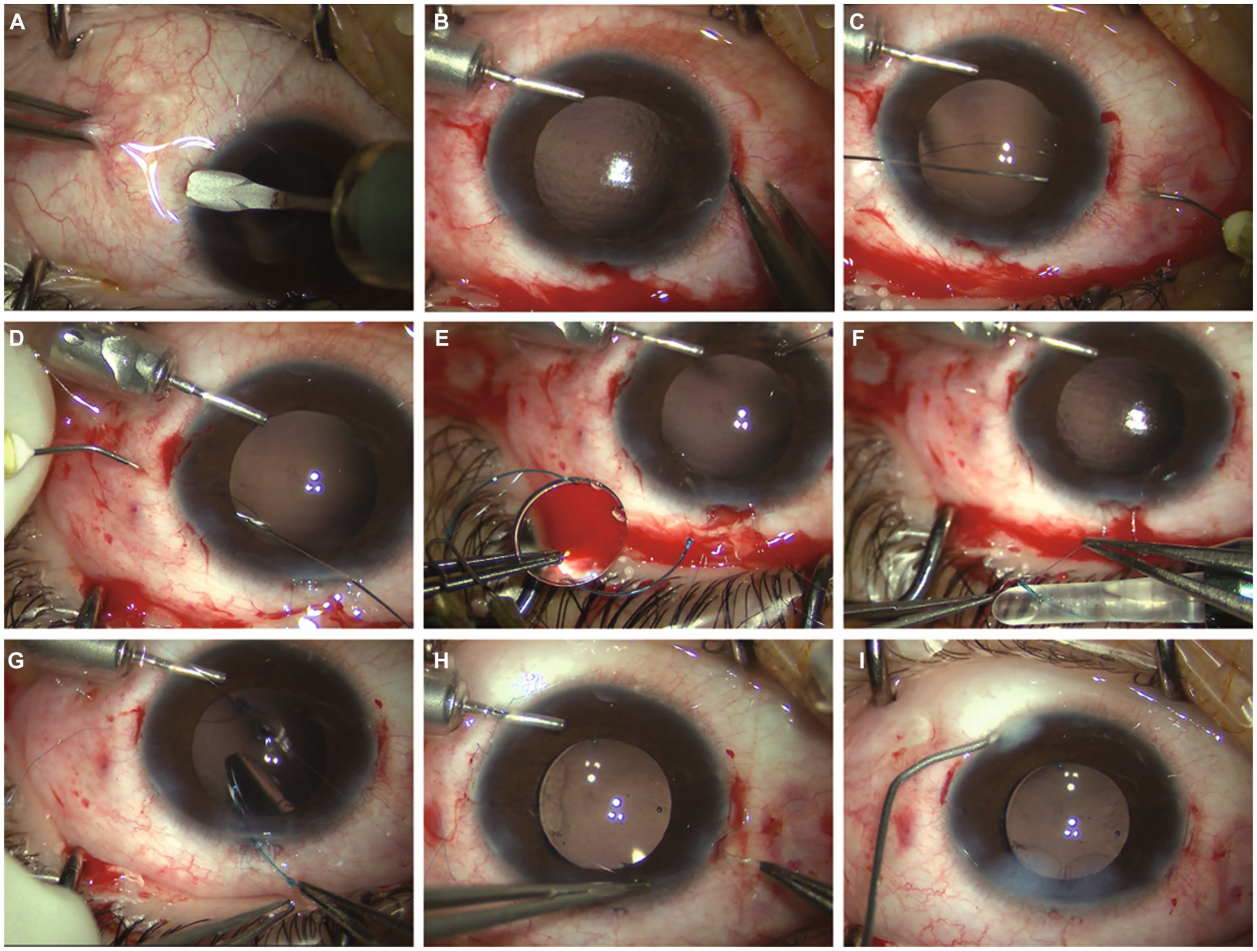
**(A)** Two scleral pockets are dissected by a crescent blade posteriorly from the limbus at 3 o’clock and 9 o’clock, achieving a thickness of 300 μm and a length of 3.0 mm. **(B)** After anterior chamber infusion, puncture points are marked at the middle line of the pocket beds 2.0 mm posterior to the limbus on both sides. **(C)** The straight needle of a double-armed 9–0 polypropylene suture is introduced at one puncture point at 9 o’clock through the conjunctiva and full thickness of the scleral pocket, passing through the anterior chamber and guided out via the side paracentesis at 2:30 o’clock. The straight needle is again passing backward through the same side paracentesis, threading through the anterior chamber and docked into the opening of a 30-G needle which is introduced at 1.0–2.0 mm adjacent to the puncture point at 9 o’clock and then removed externally. **(D)** The same manipulations are performed at the other puncture point at 3 o’clock, leaving two suture-loops externally through the superior incision. **(E)** The two haptic ends of the intraocular lens (IOL) are cauterized to make a flange. **(F)** The leading haptic of the IOL is pushed out of the cartridge and the suture loop at 9 o’clock is knotting at the junction of the enlarged flange. **(G)** The optic of the folded IOL is injected into the anterior chamber, the trailing haptic left externally is tied with the same knot using the double sutures with its loop at 3 o’clock. **(H)** The two pairs of suture ends are retrieved through the scleral pocket opening pulled out by the Sinskey hook and knotted by tension adjustable knot to center the optic of the IOL. **(I)** The superior incision is sutured with 10–0 threads and the side incisions are watertight.

**Figure 2:**
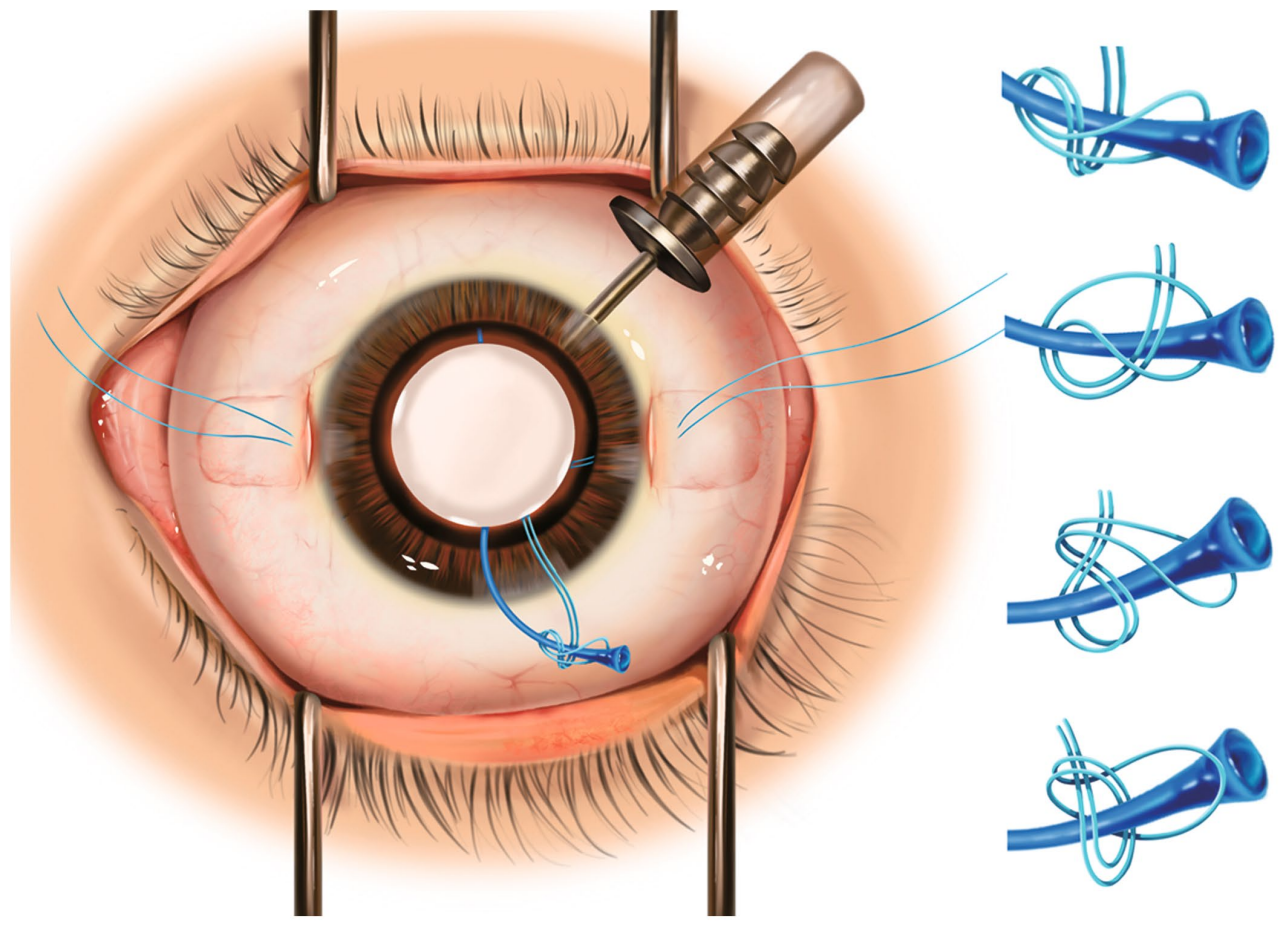
Left: Schematic figure demonstrating intrascleral fixation of flanged three-piece foldable intraocular lens with Hoffman pocket technique and special knots. Right: Four different types of wrapping methods for the knots.

Two scleral pockets are created by a crescent blade posteriorly from the limbus at 3 o’clock and 9 o’clock. The pockets are extended perpendicular to the limbus and continued for 3.0 mm, keeping a uniform depth of 300 μm in the sclera (Figure 1A). Using a 20-G microvitreoretinal blade, a paracentesis is made at 4 o’clock for 20-G infusion, and two side paracenteses are made at 2:30 o’clock and 9:30 o’clock for sutures. A superior corneal incision is made using a 3.0 mm sharp-tip keratome. Subsequently, two puncture points are marked by calipers at the pocket beds’ midline and 2.0 mm posterior to the limbus on both sides (Figure 1B). A double-armed 9–0 polypropylene suture (Mani, Tochigi, Tokyo, Japan) with one straight needle and one curved needle is used for IOL fixation. The straight needle is introduced at one puncture point at 9 o’clock through the conjunctiva and full thickness of the scleral pocket, passing through the anterior chamber and guided out via the side paracentesis at 2:30 o’clock with the assistance of a vitreoretinal forceps. Afterwards, the straight needle is again passed back through the same side paracentesis, threading through the anterior chamber and docked into the opening of a 30-G needle which is introduced at 1.0–2.0 mm adjacent to the puncture point at 9 o’clock and then removed externally (Figure 1C). The same manipulations are performed at the other puncture point at 3 o’clock. Next, the two suture-loops are dragged externally from the anterior chamber out of the superior incision by the forceps for IOL fixation (Figure 1D). Following this, the two haptic ends of the IOL are cauterized to create a flange (Figure 1E). The PCIOL is loaded in the cartridge and the leading haptic is pushed out. Then, the double sutures with its loop at 9 o’clock are wrapped around the flanged end of the leading haptic, passing behind the standing part, and the flanged end is tucked into the suture-loop, which is finally tightened to form a knot at the junction of the enlarged flange (Figure 1F). We illustrate four types of wrapping methods in Figure 2. After the leading haptic tied with the double sutures and the optic of the folded IOL is injected into the anterior chamber, the trailing haptic left externally is tied with the same knot using the double sutures with its loop at 3 o’clock (Figure 1G), and then pushed subsequently into the eye using a Sinskey hook. Thereafter, each suture end is retrieved through the scleral pocket opening by placing the Sinskey hook into the pocket and externalized after adjusting the suture tensions on both sides for the position of the PCIOL. The two pairs of suture ends are tied by tension adjustable knot to further center the optic of the PCIOL (Figure 1H). Finally, the superior incision is sutured with 10–0 threads and the side incisions are watertight (Figure 1I). An additional movie file shows this in more details (Supplementary Video 1).

All surgeries were performed by a singular experienced surgeon (PQ.Z), under general anesthesia for patients under 13 years old and retrobulbar anesthesia for the remaining. For cases with silicone oil retention, a complete pars plana vitrectomy (PPV) was performed to remove remnant silicone oil droplets prior to IOL fixation. Lensectomy was conducted beforehand to retrieve subluxated lenses or disintegrated lens capsules.

### Statistical analysis

All statistic analysis was conducted using SPSS software version 26.0 for Windows (SPSS, Chicago, Illinois, USA). The Snellen VA measurements were converted to the logarithm of the minimum angle of resolution (logMAR) units for the statistical analyses. The Wilcoxon signed-rank test was used to identify the changes between the preoperative and postoperative BCVA. The changes of IOP and ECD before and after the operation were analyzed by paired *t*-test. The statistically significance was considered when *p* value was less than 0.05.

## Results

Twelve eyes of twelve patients (9 males, 3 females) were included in this study. The mean patient age was 19.3 ± 14.6 years at the time of the surgery. The lenses of seven patients had been extracted in previous surgeries due to traumatic retinal detachment (4 eyes), rhegmatogenous retinal detachment (1 eye), intraocular foreign body (1 eye), and congenital lens subluxation (1 eye). Four patients had subluxated lenses (4 eyes) and one patient (1 eye) had a dislocated IOL due to ocular trauma. The mean follow-up time was 6.50 ± 2.97 months. Detailed patient characteristics are provided in Table 1 and detailed comparison of clinical data before and after surgery are provided in Table 2. Table 3 shows that the mean preoperative BCVA was 0.8 (1.6) logMAR, and improved to 0.45 (0.8) logMAR at the last visit (*p* = 0.012). Differences in IOP and ECD before and after surgery were, respectively, not statistically significant (*p* = 0.395 and *p* = 0.813) and generally remained in the normal range postoperatively. One pediatric patient with congenital lens subluxation experienced recurrent pupillary capture of the IOL optic as well as increased IOP during the follow-up period. Using a mydriatic agent, the patient’s IOL capture was relieved, yet recurred after terminating medication. Ultimately, the IOL was repositioned using a 30-gauge needle under the slit lamp and a miotic agent was applied to prevent recurrence. IOP-lowering eye drops were used during this period and helped maintaining the IOP between 22 and 24 mmHg and were discontinued after the IOP returned to normal. No other intraoperative or postoperative complications were recorded till the last follow-up visit.

**Table 1:** Individual patient characteristics.

Case	Gender	Age	Laterality	Axial Length	Indication for surgery	Ophthalmolmic comorbidities	Follow-up time
		years		mm			months
1	Male	10	Right	21.37	Postoperative aphakia	Traumatic RD	12
2	Male	29	Right	25.16	Traumatic aphakia	Traumatic lens subluxation	8
3	Female	5	Right	20.95	Postoperative aphakia	Traumatic RD	8
4	Female	14	Right	24.00	Postoperative aphakia	Traumatic RD	11
5	Male	11	Left	25.23	Postoperative aphakia	Intraocular foreign body	6
6	Male	11	Right	22.48	Traumatic aphakia	Traumatic lens subluxation	5
7	Male	15	Left	22.30	Postoperative aphakia	Traumatic RD; Stickler syndrome	3
8	Male	35	Right	23.42	Traumatic aphakia	Traumatic lens subluxation	3
9	Male	11	Right	26.52	Traumatic IOL dislocation	Congenital cataract	3
10	Male	13	Left	24.71	Postoperative aphakia	Congenital lens subluxation	5
11	Male	21	Left	23.21	Postoperative aphakia	RRD	7
12	Male	57	Right	23.30	Traumatic aphakia	Traumatic lens subluxation	7

RD, retinal detachment; IOL, intraocular lens; RRD, rhegmatogenous retinal detachment.

**Table 2:** Comparison of clinical data before and after surgery.

Case	BCVA, logMAR		IOP, mmHg		ECD, cells/mm^3^		Postoperative complications
	Pre	Post	Pre	Post	Pre	Post	
1	2.0	1.0	7.2	8.1	2,242	2,618	None
2	0.3	0.1	Tn	12.9	Unmeasurable	2,359	None
3	1.9	1.8	Tn	17.8	Unmeasurable	2,844	None
4	1.9	0.5	18.1	18.8	3,120	3,081	None
5	0.6	0.5	14.1	19.9	2,278	2,205	None
6	2.0	0.4	20.8	16.7	2,819	2,885	None
7	1.0	1.2	13.6	20.3	3,393	3,303	None
8	0.5	0.3	29.8	20.5	2,700	2,658	None
9	0.4	0.2	27.5	15.2	2,850	2,969	None
10	0.4	0.2	24.1	20.2	3,153	3,826	Pupillary IOL capture
11	2.0	0.9	14.3	16.8	2,513	2,507	None
12	0.2	0.2	14.8	10.3	2,339	1,628	None

BCVA, best-corrected visual acuity; IOP, intraocular pressure; ECD, endothelial cell density; IOL, intraocular lens.

**Table 3:** Analysis of clinical data before and after surgery.

Preoperative BCVA (logMAR)	
M (IQR)	0.8 (1.6)
Postoperative BCVA (logMAR)	
M (IQR)	0.45 (0.8)
P	0.012*
Preoperative IOP (mmHg)	
MD ± SD	18.43 ± 7.05
Postoperative IOP (mmHg)	
MD ± SD	16.46 ± 4.12
P	0.395†
Preoperative ECD (cells/mm^**3**^**)**	
MD ± SD	2740.70 ± 399.32
Preoperative ECD (cells/mm^**3**^**)**	
MD ± SD	2740.25 ± 559.28
P	0.813†

*Wilcoxon signed-rank test for the analysis of BCVA.

†Paired *t*-test for the analysis of IOP and ECD.

BCVA, best-corrected visual acuity; IOP, intraocular pressure; ECD, endothelial cell density; P < 0.05 is marked in bold.

## Discussion

Application of foldable three-piece IOLs have been reported in a handful of sutureless scleral fixation techniques (2, 8). Given that suture fixation of PCIOLs remains the most widely accepted method for significant long-term stability and safety, we modified it with a special designed foldable three-piece PCIOL in our previous study (7). The haptic ends of this PCIOL (PY60AD, HOYA Medicals, Shinjuku, Tokyo, Japan) is designed with an enlarged cone shape, which enables us to knot the suture directly at the junction of haptic ends and avoid suture slippage. Based on this, we intended to improve upon this technique and broaden the usage of three-piece IOLs of other commonly designed varieties. The present study describes a modified suture scleral fixation approach combining Hoffman pocket technique and flanged IOL fixation technique, with a special knot. Clinical observation during the follow-up period reported improved BCVA without severe intraoperative and postoperative complications.

There are several advantages of to this technique. First, the scleral pocket technique eliminates the need for, and associated risks of, conjunctival dissection, scleral cauterization, and sutured wound closure. By bypassing these procedures, we posit to enhance patients’ recovery and comfort. Additionally, it is also easier to perform in the distal location rather than via a triangular flap. More importantly, burying the suture knot in the pocket mitigates the risks of overlying conjunctiva erosion, subsequent endophthalmitis, and suture breakage (9). Second, previous studies involving sutured scleral fixation of foldable three-piece IOLs generally involve dialing a hole in the optic of the IOL (10). Our approach of creating a flange not only effectively prevents the scleral suture knots from becoming loose at haptic ends, but also enables flexible application of various types of foldable three-piece PCIOLs. Another noteworthy aspect is the flexibility of performing the knotting procedure, which can be accomplished with at least four different wrapping methods at the junction of the haptic ends (Figure 2). We note that this method is much easier for surgeons to master without the need to split the suture loop in half. Moreover, our technique streamlines surgical maneuvers and expedites the entire procedure for dislocated IOLs by eliminating the need to explant the posteriorly dislocated IOL via creating a new incision. This step, commonly associated with complications such as corneal injury, unstable IOP, and postoperative astigmatism, is thereby circumvented. Further, our approach strengthens the existing internal scleral fixation methods for repositioning dislocated IOLs within a closed-eye system, specifically designed for foldable three-piece IOLs (3, 4). Notably, compared to Yamane’s method (2), our technique may be a more stable choice for patients with thinner sclera, such as pediatric or high myopic patients, as well as ones with scleral scarring due to PPV incisions, because of the potential sliding of flanged haptics under those conditions.

There are several important considerations to address during the surgical procedures. 1) For the main incision, it is feasible to opt for either a clear corneal incision or a scleral tunnel incision. 2) We recommend making scleral pockets before the three ports for PPV, as pre-existing PPV incisions may occupy the space required for the pockets. 3) Anterior and posterior infusion are both viable options, depending on specific surgical requirements for individual cases. 4) What we particularly emphasize is the necessity of creating a haptic flange. While some previous techniques have reported using a suture loop to secure the haptics without a flange for repositioning dislocated IOLs (11, 12), we observed postoperative suture slippage in two cases involving foldable three-piece IOLs where haptic flanges were not created (not reported in this study). 5) Additionally, it is worth noting that our technique is not suitable for IOLs with haptics made from polyimide, as the material cannot be cauterized.

Concerning postoperative complications, the patient who exhibited recurrent pupillary capture of the IOL optic, was suspected to have Marfan Syndrome (MFS) based on his ocular manifestations, while the parents of the patient declined gene test for a definitive diagnosis. A detailed examination of the patient’s peripheral retina revealed lattice degeneration, which was subsequently lasered during PPV. In pseudophakic patients with MFS, it had been reported that myopathy affecting pupil constrictors and dilators can result in pliable iris and reverse pupillary block, which may induce a higher rate of pupillary capture (13). In such cases, we recommend relocating the IOL fixation plane from 2.0 to 2.5 mm posterior to the limbus, along with peripheral iridectomy. For patients with irreversible recurrent IOL capture, pupillary cerclage suturing is also a viable option.

In conclusion, this surgical technique innovatively combines the Hoffman pocket, flanged IOL, and knotting techniques, to enable flexible usage of three-piece IOLs with secure haptic fixation and achievement of improved visual outcomes with fewer complications. Future studies with larger sample sizes and extended follow-up are needed to validate long-term functional and anatomic outcomes.

## Data availability statement

The raw data supporting the conclusions of this article will be made available by the authors, without undue reservation.

## Ethics statement

The studies involving humans were approved by Ethical Review Board of Xinhua Hospital Affiliated to Shanghai Jiao Tong University School of Medicine. The studies were conducted in accordance with the local legislation and institutional requirements. Written informed consent for participation was not required from the participants or the participants’ legal guardians/next of kin in accordance with the national legislation and institutional requirements. The need for participation consent was waived due to the retrospective nature of this.

## Author contributions

HY: Conceptualization, Data curation, Formal analysis, Funding acquisition, Investigation, Methodology, Project administration, Resources, Software, Supervision, Validation, Visualization, Writing – original draft, Writing – review & editing. MW: Conceptualization, Data curation, Formal analysis, Funding acquisition, Investigation, Methodology, Project administration, Resources, Software, Supervision, Validation, Visualization, Writing – original draft, Writing – review & editing. WS: Conceptualization, Data curation, Formal analysis, Funding acquisition, Investigation, Methodology, Project administration, Resources, Software, Supervision, Validation, Visualization, Writing – original draft, Writing – review & editing. JL: Conceptualization, Investigation, Methodology, Project administration, Resources, Validation, Writing – review & editing. YX: Conceptualization, Data curation, Investigation, Methodology, Resources, Supervision, Validation, Visualization, Writing – review & editing. PF: Conceptualization, Investigation, Methodology, Project administration, Resources, Validation, Visualization, Writing – review & editing. JP: Methodology, Project administration, Validation, Visualization, Writing – review & editing. HJ: Conceptualization, Investigation, Methodology, Validation, Visualization, Writing – review & editing. PZ: Conceptualization, Data curation, Formal analysis, Funding acquisition, Investigation, Methodology, Project administration, Resources, Software, Supervision, Validation, Visualization, Writing – original draft, Writing – review & editing.

## Supplementary material

Supplementary Video 1 The surgical procedures of the technique.
